# Vibrotactile-Based Rehabilitation on Balance and Gait in Patients with Neurological Diseases: A Systematic Review and Metanalysis

**DOI:** 10.3390/brainsci11040518

**Published:** 2021-04-19

**Authors:** Sara De Angelis, Alessandro Antonio Princi, Fulvio Dal Farra, Giovanni Morone, Carlo Caltagirone, Marco Tramontano

**Affiliations:** 1 Fondazione Santa Lucia IRCCS, 00179 Rome, Italy; s.deangelis@hsantalucia.it (S.D.A.); a.princi@hsantalucia.it (A.A.P.); g.morone@hsantalucia.it (G.M.); c.caltagirone@hsantalucia.it (C.C.); 2 SOMA-Istituto Osteopatia Milano, 20126 Milan, Italy; fulviodalfarra@outlook.it; 3 Department of Movement, Human and Health Sciences, University of Rome “Foro Italico”, 00185 Rome, Italy

**Keywords:** balance rehabilitation, gait rehabilitation, neurological disease, cerebrovascular disease, motor-cognitive, vibrotactile feedback

## Abstract

Postural instability and fear of falling represent two major causes of decreased mobility and quality of life in cerebrovascular and neurologic diseases. In recent years, rehabilitation strategies were carried out considering a combined sensorimotor intervention and an active involvement of the patients during the rehabilitation sessions. Accordingly, new technological devices and paradigms have been developed to increase the effectiveness of rehabilitation by integrating multisensory information and augmented feedback promoting the involvement of the cognitive paradigm in neurorehabilitation. In this context, the vibrotactile feedback (VF) could represent a peripheral therapeutic input, in order to provide spatial proprioceptive information to guide the patient during task-oriented exercises. The present systematic review and metanalysis aimed to explore the effectiveness of the VF on balance and gait rehabilitation in patients with neurological and cerebrovascular diseases. A total of 18 studies met the inclusion criteria and were included. Due to the lack of high-quality studies and heterogeneity of treatments protocols, clinical practice recommendations on the efficacy of VF cannot be made. Results show that VF-based intervention could be a safe complementary sensory-motor approach for balance and gait rehabilitation in patients with neurological and cerebrovascular diseases. More high-quality randomized controlled trials are needed.

## 1. Introduction

Balance is a complex multi-factorial system in which both motor and sensory components interact one with other [1]. The central nervous system integrates the information originate from visual, vestibular, proprioceptive and cognitive systems in a continuous sensorial re-weighting that ensure postural control in static and dynamic condition [1]. The weighting of the sensory inputs depends on both the environmental conditions and the motor task performed by the subject [2,3,4].

The integration of multi-sensory information is impaired in neurological diseases [4,5,6,7], leading to balance and postural control disorders and consequently to a high prevalence of falls and fear of falling [8]. It is known that among stroke survivors about 73% are reported to be fallers, 45–68% of people with Parkinson’s disease (PD) fall each year and that up to 50% of the Multiple Sclerosis (MS) population are estimated to be fallers [8,9]. Postural instability and fear of falling represent one of the major causes of decreased mobility, limitation in physical activity, and social isolation resulting in reduced quality of life [10]. In recent years, rehabilitation strategies have been carried out considering an active involvement of the patients during the rehabilitation sessions and a challenging task-oriented exercise [11]. 

Previous studies [12,13] have suggested that peripheral afferent input could lead to compensation of balance system impairments. Different therapeutic approaches based on the treatment of balance afferents are commonly used, including vestibular rehabilitation [7,14,15,16,17], visual training [6,18,19,20], and proprioceptive training [21,22,23]. 

Furthermore, the connection and the relationship between different aspects of cognitive and motor function is increasingly documented on motor and balance interventions as well as for the fear of falling in subjects affected by central nervous system diseases [24,25].

According to these approaches, new technological devices and paradigms have been developed to increase the effectiveness of rehabilitation by integrating multisensory information and augmented feedback [19,20,26] promoting the involvement of a top-down paradigm in neurorehabilitation, with an increase in the involvement of the cognitive functions [20,25,26,27]. 

In this context, through vibrotactile feedback (VF), the vibratory stimulus was used as a peripheral therapeutic input, in order to provide spatial proprioceptive information to guide the patient during a motor task exercise [28,29]. VF is user-friendly and usable in the clinical rehabilitation setting [30], it needs a small actuator that generates a signal and supplies vibrational stimuli [21,31]. To date, many VF devices have been used to improve gait spatiotemporal parameters, facilitating a task-oriented rehabilitative approach in patients with neurological disorders [28,31,32]. Moreover, the purpose of the cue could be variable: it may represent information for alert, direction, spatial orientation and other communication, which should be made explicit to the performer before the training start [33]. It could be used alone or in combination with other or as a cognitive-motor task [33]. Furthermore, it could be combined with devices that make it feasible to assess the motor tasks performed by the patient and to adapt the vibrator stimulus to the performance [34]; thus, during the rehabilitation session, once an exercise has been assessed as being improperly performed, the patient can be provided with feedback in order to stimulate an adaptation and the motor task improvement [29] 

Although the VF has been shown to be useful for patients with neurological diseases across a variety of situations [29,35,36], there are no quantitative and qualitative reviews that systematically report the effect of VF on the motor functions in patients with neurological disorders. The present systematic review and metanalysis aimed to explore the effectiveness of the VF on balance and gait rehabilitation in patients with neurological diseases. 

## 2. Materials and Methods

This systematic review and meta-analysis was performed in accordance with the PRISMA (Preferred Reporting Items for Systematic Reviews and Meta-Analyses) statement [37] and following the Cochrane Handbook for Systematic Reviews of Interventions [38]. The study’s protocol was registered on PROSPERO International prospective register of systematic reviews website (registration number CRD42021217837).

### 2.1. Search Strategy and Eligibility Criteria

Electronic databases searched in November 2020 were MEDLINE (PubMed), PEDro (Physiotherapy Evidence Database). Search terms used were (“balance” AND “vibrotactile” OR “haptic” AND “neurological disorde*” OR “stroke” OR “parkinson” OR “multiple sclerosis” OR “traumatic brain injury”). Search terms were modified for each database and appropriate subheadings were used for each database searched (for detailed see Appendix A). 

Controlled and non-controlled clinical trials (i.e., randomized or non-randomized trials), retrospective studies, case reports, case series, and observational studies, were included. No restrictions related to publication date, sex, and country were applied. Participants included in the studies presented static and/or dynamic balance disorders and a diagnosis of stroke, PD, MS and traumatic brain injury (TBI).

### 2.2. Study Selection and Data Collection Process

Duplicate records were identified and removed using the EndNOTE software. Study eligibility assessment and the data extraction process, carried out by two independent co-authors (SDA and AAP). In case of any disagreement, the opinion of a third author (MT) was used to reach accordance. The first selection of studies was initially conducted basing on the title and abstract and afterwards full-text articles were examined. 

The summary of results was reported following the PRISMA (Preferred Reporting Items for Systematic Reviews and Meta-Analyses) statement [37]. Two authors (SDA and AAP) independently extracted the following relevant features of the included studies: name of primary author and publication year, participants, rehabilitative intervention, outcome measures. 

The methodological quality of evidence was assessed using the PEDro scale [39] for the controlled trials and using a modified version of the Newcastle-Ottawa Scale (NOS) [14,40] for the observational studies. The assessment was performed by two authors (SDA and AAP); discrepancies were resolved by consensus with a third reviewer (MT) as an arbiter. The PEDro scale ranging from 0 to 10, the modified NOS ranging from 0 to 7. In both scales, the maximum score shows a better methodological quality. 

### 2.3. Measures and Synthesis of Results

Data concerning qualitative synthesis are reported descriptively by using means, DS, percentages and ranges. A meta-analysis was performed using “Review Manager 5.3.5” (The Nordic Cochrane Centre, København, Dänemark, https://revman.cochrane.org/#/myReviews. Only RCTs investigating comparable outcome measures were included in this quantitative synthesis. Alpha level was set at 0.05 to test for overall effect. For continuous measures (“pitch and roll” sway and angular velocity), standardized mean difference (SMD or Hedges’s g) with 95% CI was obtained by using a random-effects model, in reason of the clinical and methodological variability detected among included studies. When available, absolute values (degrees for angles, degrees/seconds for angular velocity) were used; on the contrary, percentages were considered. An effect size ranging from 0.2 to 0.49 is to consider “small”, from 0.5 to 0.79 “moderate” and a score of 0.8 or above represents a large effect. Heterogeneity was measured through I^2^ statistics and explains how much of the variability among studies is due to heterogeneity rather than to chance. Values included in the range 0–40% may imply “no important” heterogeneity, 30–60% suggest “moderate” levels, 50–90% could indicate “substantial” and 75–100% “considerable” levels.

## 3. Results

**Figure 1:**
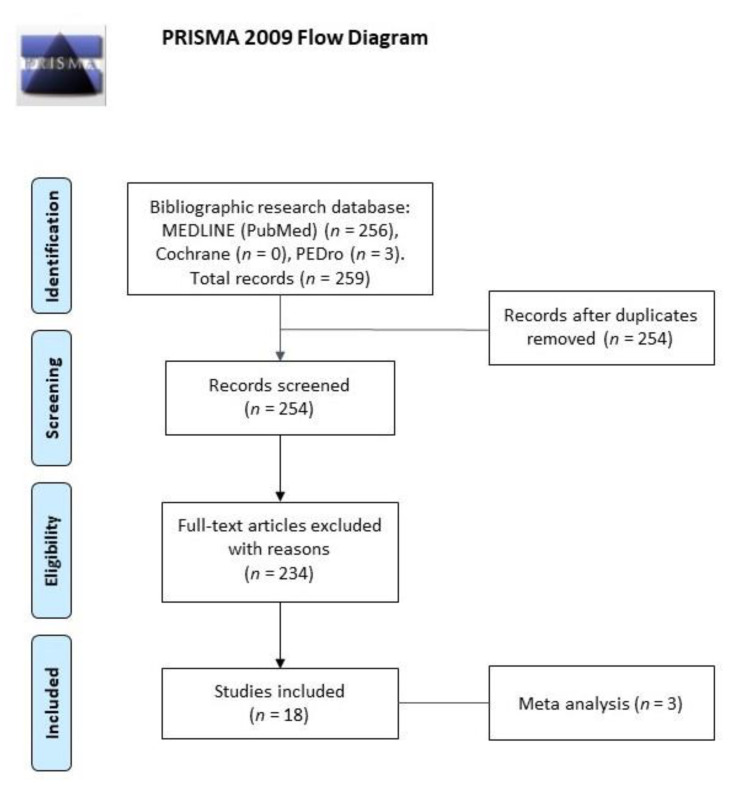
Prisma flow diagram.

Electronic searches identified 259 studies. Titles and abstracts were examined according to inclusion and exclusion criteria. The full texts of the articles were read to determine the eligibility. Furthermore, reference lists of identified articles were screened for additional relevant literature. Comparison of the retrieved titles identified five studies that were duplicates. The result consisted of 254 articles eligible for inclusion. After a full-text analysis 234 articles were excluded due to the following reasons: (a) did not concern balance and/or gait rehabilitation; or (b) did not provide active vibratory feedback. A total of 18 studies met the inclusion criteria and were included, just once, in the present systematic review (Figure 1). 

Table 1 presents a narrative summary of results included studies with their associated characteristics and patient features. In particular, the following data are reported: first author’s name, publication year, participants, intervention, and outcome measures.

The included studies were all published in English and were conducted in different countries. Six studies came from the USA, three from the Republic of Korea, and three from Japan; two studies were carried out in Germany, two in Switzerland, and two in The Netherlands; Italy, Spain, and China have contributed to this review with one study each one. Of the four investigated neurological diseases, 11 studies (61.1%) included patients with a diagnosis of PD; 6 studies (33.3%) concerning patients with stroke, specifically three studies included chronic stroke patients (50%), two studies subacute-stroke patients (33,3%) and one study (16.6%) reported no data concerning the onset time; one study (5.5%) involved patients with a diagnosis of MS an and no studies including patients with TBI. 

A total of 344 patients, of which there were 192 with neurological disease and presence of static and/or dynamic balance disorders, were included in the review. There were 127 participants with a clinical diagnosis of PD, 55 strokes, and 10 MS.

The Modified NOS scale was used to assess the quality of no RCTs. The NOS scale of the included studies ranged between 4 and 6 with a mean score of 5.36 points out of 7 (Table 2). The PEDro score assessing the risk of bias of the included RCTs ranged between 4 and 7, showing two different levels of quality: high-quality studies (= PEDro score 6–10) and fair quality studies (= PEDro score 4–5). The mean PEDro score was 5.25 points out of 10 (Table 3). None of the included studies reached the maximum score neither in Modified NOS nor in the PEDro scale mostly because of the participants’ selection (low number and non-randomized). 

The primary aim of the included studies was to evaluate the effect of VF on balance and gait in patients with static and dynamic postural impairments. The vibratory stimulus was provided as feedback to facilitate the patient’s movement during static and dynamic tasks. The vibrotactile was supply both as unique augmented feedback or combined with others (haptic feedback different to the vibratory one), during different balance and gait tasks and using various devices and software. Moreover, not only one body application was used. Vibrotactile effectors were used mounted on belts or directly in contact with the patient’s skin and placed in different body areas: head [35,41,42], sternum [43], waist level [44], at L4/L5 level [45,46,47], hip [48,49]; lower limb [50,51,52], anklets [32] and foot [53]. The experimental interventions lasted several days (maximum of 4 weeks) [54] or one single day and were mostly conducted in a hospital or laboratory setting. One study was carried out in a home situation [43]. Concerning the outcomes, both instrumental and clinical assessment were performed. The instrumental ones analyzed the static and dynamic parameters of postural control, while various clinical-scale tests and questionnaires were used to clinically assess balance, gait, risk of fall and the patient’s self-assessment of balance disorders and satisfaction in the intervention. All the outcomes are detailed in Table 1. 

### 3.1. VF Effects on Pitch Sway Angular Velocity

**Figure 2:**
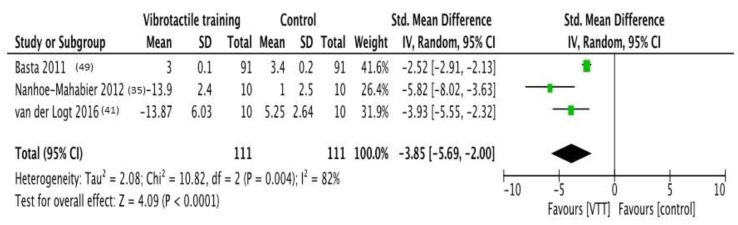
**Forest plot of comparison:** Overall effect of Vibrotactile feedback training in comparison to no-feedback interventions. Outcome: pitch sway angular velocity. Abbreviations: VTT, vibrotactile training; CI, confidence interval; SD, standard deviation [35,41,49].

Three trials [35,41,49] were included in the analysis, with a sample size of 111. Forest plot indicates that VF resulted statistically significant in all these studies, when compared to control group. There was an overall large effect in favor of VF (SMD = −3.85 [−5.69; −2.00]); *p* < 0.0001; Heterogeneity is substantial and significant (I^2^ = 82%; *p* = 0.004). The forest plot of comparison is shown in Figure 2. 

### 3.2. VF Effects on Pitch Sway Angle

**Figure 3:**
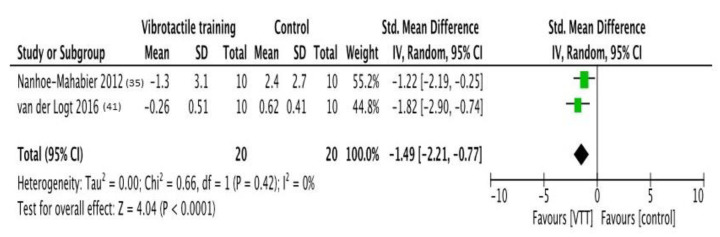
**Forest plot of comparison:** Overall effect of Vibrotactile feedback training in comparison to no-feedback interventions. Outcome: pitch sway angle. Abbreviations: VTT, vibrotactile training; CI, confidence interval; SD, standard deviation [35,41].

Two studies [35,41] were considered, with an overall sample of 20 subjects. The aggregate analysis shows significative effects for VF in both trials. Effect size is estimated as −1.49 (−2.21; −0.77), *p* < 0.0001; Heterogeneity is absent (I^2^ = 0%) and not significative (*p* = 0.42). The forest plot of comparison is shown in Figure 3.

### 3.3. VF Effects on Roll Sway Angular Velocity

**Figure 4:**
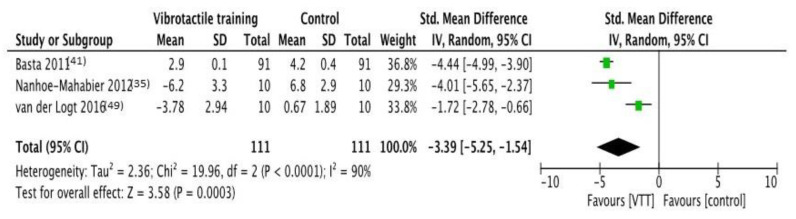
**Forest plot of comparison:** Overall effect of Vibrotactile feedback training in comparison to no-feedback interventions. Outcome: roll sway angular velocity. Abbreviations: VTT, vibrotactile training; CI, confidence interval; SD, standard deviation [35,41,49].

The analysis included three trials [35,41,49] (sample size: 111), showing significant effects in favor of VF if compared to no feedback interventions. The overall effect size is estimated as large (−3.39 [−5.25; −1.54] *p* = 0.0003) and considerable levels of heterogeneity (I^2^= 90%, *p* < 0.0001) were detected. The forest plot of comparison is shown in Figure 4.

### 3.4. VF Effects on Roll Sway Angle 

**Figure 5:**
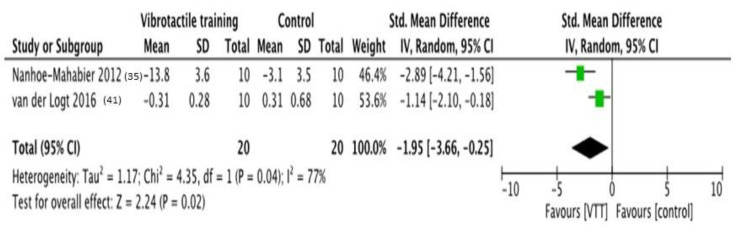
**Forest plot of comparison:** Overall effect of Vibrotactile feedback training in comparison to no-feedback interventions. Outcome: roll sway angle. Abbreviations: VTT, vibrotactile training; CI, confidence interval; SD, standard deviation [35,41].

Two RCTs [35,41] were included in this analysis, considering 20 subjects. Forest plot shows how both studies support the superiority of VTT in comparison to control treatments. The effect size is −1.95 (−3.66; −0.25) *p* = 0.02, and heterogeneity is substantial and significative (I^2^ = 77%; *p* = 0.04). The forest plot of comparison is shown in Figure 5.

**Table 1:** Description of included studies.

	Participants	Intervention	Outcome Measures
Rossi, 2020 [32]	*n* = 27 (PD = 15; Healthy = 12) University laboratory Inclusion criteria: PD diagnosis Time since diagnosis onset (yr): 9.0 ± 4.9 Age (yr): PD (61 ± 7.3); Healthy (93.7 ± 13.6)	Participants were asked to walk in three different sensorial condition: - no-haptic suggestions mode: walked while no haptic suggestion was sent; - haptic-suggestions mode: as in the previously condition with the haptic stimuli switched on; - mixed mode: half walking path with stimuli and half without. Frequency: 1 time for 1 day	Step’s elevation and length; Velocity and inter-stride variance; FOG time.
Kodama, 2019 [34]	*n* = 9 (Stroke = 9) Hospital ward Inclusion criteria: Stroke: age 50–80 years, stroke > 6 months ago, completion of conventional therapy, stand up unsupported for 10 min, sense BF vibrations. Time since diagnosis onset (yr): 6.6 ± 3.6 Age (yr): 81.56 ± 44	Two task-oriented balance training exercises: - standing on a rubber foam mat: participants stood barefoot on the mat with their eyes open and were instructed to use the BF information to stabilize their postural sway; -weight shifting to the paralyzed limb: participants were instructed to move their paralyzed lower limb forward and then put their weight on that limb. Frequency: 2 times a week for 2 weeks.	CoP A/P CoP M/L
Afzal, 2018 [50]	*n* = 10 (Stroke = 10) University hospital ward Inclusion criteria: subacute phase, able to walk 10m without assistance, Brunnstrom stage ≥ 3 Time since diagnosis onset (d): 62.5 ± 26.6 Age (yr): 57.7 ± 10.6	Participants were asked to walk for 10 m in four different trial conditions: - Normal walk in a straight line without any assistance/cue. Subject maintains self-preferred walking speed an operator calculates the normal gait speed of the subject; - Walk with only tactile cue. Subject maintains self-preferred walking speed and operator calculates the gait speed; - Walk with only kinesthetic cue. Speed is set to normal gait speed + 0%, + 20% and + 40% in separate trials; - Walk with both tactile cue and kinesthetic cue. Speed is set to normal gait speed + 0% + 20% and + 40% in separate trials. Each trial condition was performed two times by the participants.	Gait speed (m/s) RMS of ML Tilt (degrees) Stance Symmetry Ratio - % muscle activity EMG
Fung, 2018 [45]	*n* = 10 (PD = 10) University laboratory Inclusion criteria: PD diagnosis, 2–4 H&Y, stand independently for 5 min, walk independently for 10 m. Time since diagnosis onset (yr): not declared Age (yr): PD (61 ± 7.3); Healthy (70.7 ± 7.89)	24 trials of dynamic WSBE by using the SBS’s custom application that provided visual and auditory instructions pointing out the start and end of each trial. Frequency: 1 time for 1 day	LOS (A/P & M/L) XCOR (A/P & M/L) PE (A/P & M/L) PTA (A/P & M/L)
High, 2018 [44]	*n* = 10 (PD = 9, HO = 10, ORF = 9) University laboratory Inclusion criteria: PD participants: PD diagnosis, UPDRS motor score 25.22 ± 13.24); ORF participants: at least two falls in the last year (American and British Geriatric Society classification). Time since diagnosis onset (yr): not declared Age (yr): PD (69 ± 10.25), HO (76.4 ± 6.8), ORF (82 ± 9.72)	Stay still barefoot for 30s in each of the following conditions: (1) feet together, eyes open on firm surface (2) feet together, eyes closed on firm surface (3) feet together, eyes open on foam surface (4) feet together, eyes closed on foam surface (5) tandem stance with eyes open on firm surface Frequency: 2 consecutive trials for 1 day.	Path length Velocity Sway area Alpha M/L Alpha A/P
Lee, 2018 [46]	*n* = 18 (PD = 9, HO = 9) Undeclared setting Inclusion criteria: PD diagnosis, D3 < H&Y > 4 Time since diagnosis onset (yr): not declared Age (yr): PD (67.1 ± 6.5), HO (67.7 ± 6.9)	12 familiarization trials to acclimate themselves to vibrotactile biofeedback; 5 min seated rest; 20 randomized trials of dynamic weight-shifting balance exercises as a function of the coding scheme and movement direction. Frequency: 1 time for 1 day	LOS (A/P & M/L) XCOR (A/P & M/L) PE (A/P & M/L)
Yasuda, 2018 [54]	*n* = 9 (Stroke = 9) Hospital ward Inclusion criteria: history of chronic stroke, age 50–80 years, stroke > 6 months ago, completion of conventional therapy, stand up unsupported for 10 min, sense BF vibrations. Time since diagnosis onset (mth): >6 Age (yr): 64.4 ± 9.2	Two task-oriented balance training exercises were used: - standing on a rubber foam mat: participants stood barefoot on the mat with their eyes open and were instructed to use the BF information to stabilize their postural sway - weight shifting to the paralyzed limb: participants were instructed to move their paralyzed lower limb forward and then put their weight on that limb. Each training session comprised 10 repetitions of the balance task (1 min per repetition, 10 min total) with a short interval between repetitions. Frequency: 2 times a week for 2 weeks.	CoP pressure data Berg Balance Scale (BBS) Functional Reach Test (FRT) Timed-Up and Go Test (TUG)
Van Wegen, 2018 [43]	*n* = 15 (PD = 15) Home situation Inclusion criteria: PD diagnosis, 1–3 H&Y, score ≥ 2 on item 28 of UPDRS, correctable postural abnormality, sufficient cognitive function, absence of relevant comorbidities, stable medication regimen. Time since diagnosis onset (yr): 8.6 ± 4.8 Age (yr): PD (70.1 ± 8.7)	In the intervention period (week 2) the UpRight was active. Two trained assessors instructed the patients that they should consciously correct their posture in response to the sensory-feedback signal. Frequency: 2 weeks	Average trunk angle in the sagittal plane. Self-reported patient satisfaction to determine feasibility and user-friendliness of the UpRight.
Afzal, 2017 [51]	*n* = 6 (Stroke = 6) Undeclared setting Inclusion criteria: No limitations in joint range of motion and sensorial feedback abilities or other diagnosed neurologic or musculoskeletal disease Time since diagnosis onset (d): 69.7 ± 24.9 Age (yr): 55.0 ± 11.0	Subjects walked 10 m distance two times in each trial. Kinesthetic stimuli were moved with the operator’s set velocity and provide a constant vibration on the skank during the swing phase.	RMS ML Stance ratio Muscle activity EMG
Yasuda, 2017 [55]	*n* = 17 (Stroke = 17) Rehabilitation center Inclusion criteria: stroke history, sufficient communication abilities, Brunsnstrom recovery Stage III, MMSE > 20, maintain balance in bipedal stance on a foam rubber mat for > 30 s, sense vibration of the BF. Time since diagnosis onset (d): 1144.94 ± 1451.63. Age (yr): 60.8 ± 17.3	One familiarization session. The BF session comprised five repetitions of the balance task (15s each), with an interval of 1 min between each repetition. Frequency: 1 time for 1 day.	CoP spatial variability. Mean velocity of CoP displacement (mm/s) Mean CoP A/P and M/L distance.
Otis, 2016 [53]	*n* = 21 (12 PD, 9 Healthy) University laboratory Inclusion criteria: PD diagnosis, physically active, without musculoskeletal or other neurological disorders. Time since diagnosis onset: not declared Age (yr): PD (67.9 ± 10.0), Healthy (66.8 ± 8.0)	Firstly, the subject was asked to walk along a corridor by performing the TUG test without cueing. Secondly, participants performed two trials under vibratory stimulation condition at 10% above baseline cadence over each type of soil (concrete, parquet, broken stone, sand, carpet living room, and carpet foam) for a total of twenty-four trials for the two conditions. Frequency: 1 time for 1 day	TUG Risk of falling
van, der Logt 2016 [41]	*n* = 10 (MS = 10) Undeclared setting Inclusion criteria: MS diagnosis, able to walk without aids, without orthopedic problems or other diseases/disabilities than MS that could affect balance. Time since diagnosis onset: not declared Age (yr): 46.8 ± 7.7	Assessment and training sequences consisting of stance and gait task while without shoes. Patients performed the assessment sequence three times and the training sequence of trial protocols once on the same day with sufficient breaks between sequences to avoid fatigue. Assessment sequence: 12 tasks, training sequence execute 3 consecutive times (7 tasks). Frequency: 1 time for 1 day	standing on one leg with eyes open standing on two legs with eyes closed standing on two legs with eyes open on foam standing on one leg with eyes open on foam standing on two legs eyes closed on foam tandem stance with eyes open and closed walking eight tandem steps with eyes open and closed - walk over a set of low (24 cm) barriers spaced one meter apart - walked eight meters with eyes open - three meters with eyes closed
Afzal, 2015 [52]	*n* = 9 (Stroke = 4, Healthy = 5) Undeclared setting Inclusion criteria: Stroke patients Time since diagnosis onset: undeclared Age (yr): (Stroke = 67.2 ± 5.5, Healthy = 26.2 ± 3.2)	The distance of the walking trial was 10 m for healthy and 6 m for stroke. The subject was asked to walk in three scenarios: normal walk, walk whit stance time matching constant vibration mode and with swing phase constant vibration mode.	Symmetry ratio M/L tilt-RMS M/L acceleration-RMS Right stance Left stance Gait speed
Lee, 2015 [47]	*n* = 20 (PD = 11, Healthy = 9) Clinical setting Inclusion criteria: PD diagnosis, 3–4 H&Y Time since diagnosis onset: not declared Age (yr): PD (70.0 ± 8.1); Healthy (67.8 ± 6.6)	All participants performed 12 familiarization trials (i.e., 3 modalities × 2 directions × 2 repetitions) to acclimate to the guidance modalities (visual, vibrotactile, and simultaneous visual and vibrotactile biofeedback) during dynamic weight-shifting balance exercises. After the completion of the familiarization trials, all participants were provided a 5 min seated rest. During the experimental session, all participants performed dynamic weight-shifting balance exercises as a function of the modality and direction with 5 repetitions for a total of 30 trials (i.e., 3 modalities × 2 directions × 5 repetitions). The order of trials was randomized for each participant. Frequency: 1 time for 1 day.	LOS (A/P & M/L) SOT score
Lee, 2013 [42]	*n* = 44 (Mild PD = 20, Advanced PD = 7; Healthy = 17) Undeclared setting Inclusion criteria: PD diagnosis, able to stand unaided, 1–3 H&Y Time since diagnosis onset: not declared Age (yr): Mild PD (67.5 ± 10.4);Advanced PD (68.6 ± 11.3);Healthy (67.5 ± 10.4)	Subjects stood on a motorized, computer-controlled platform that moved at a peak acceleration of 1.16 m/s2, a constant velocity of 0.48 m/s, and a peak deceleration of 0.58 m/s2. Thus, the stepping reaction is fairly automatic, although subjects were warned that stepping was the necessary reaction, and 3 practice trials in each direction were administered. Frequency: 1 time for 1 day	SRT step length step angular velocity number of steps total trunk displacement trunk displacement before taking the first protective step
Rossi-Izquierdo, 2013 [48]	*n* = 10 (PD = 10) Undeclared setting Inclusion criteria: 3–4 H&Y Time since diagnosis: not declared Age (yr): 67.0 (53–79 years)	A training session consisted of 5 repetitions of six selected training tasks as described above. The patient received a VF signal during training in those directions which showed a higher body sway than preset thresholds Frequency: 5 time/week for 2 weeks	Free-field body sway analysis (mobile posturography) SBDT or Gsbdt SOT DHI ABC number of falls in the least three months Comparison of the results of vibrotactile neurofeedback training with a CDP-training in PD patients
Nanhoe-Mahabier, 2012 [35]	*n* = 20 (PD = 20) Undeclared setting Inclusion criteria: PD diagnosis Time since diagnosis onset: not declared Age (yr): Feedback group (59.3±20.0); Control Group (58.6±2.5)	Real-time biofeedback during balance exercises. Frequency: 1 time for 1 day	Roll sway Pitc sway
Basta, 2011 [49]	*n* = 105 (Canal Paresis = 25), (Otolith disorder = 21), (Acustic Neuroma = 10), (Microvascualr syndrome = 12), (PD = 10), (Presbyvertigo = 13), (control group = 14) Undeclared setting Inclusion criteria: pathologic body sway at the SBDT or gSBDT tests. Time since diagnosis onset: not declared Age (yr): (Canal Paresis = 60.2 ± 13.6), (Otolith disorder = 54.6 ± 13.8), (Acustic Neuroma = 60.2 ± 10.1), (Microvascualr syndrome = 52.0 ± 10.8), PD (68.1 ± 9.1), Presbyvertigo (73.4 ± 6.0).	Vestibular rehabilitation exercise with Vertiguard training device. Frequency: 5 time/week for 2 weeks	SOT DHI VSS Pitch and Roll

A/P = anterior-posterior; ABC = activities specific balance confidence scale; BF = Biofeedback; CDP = Computerized Dynamic Post urography; CoP = Center of pressure; d = days; DHI = Dizziness handicap inventory; EMG = Electromyography; FOG = Freezing of gait; H&Y = Hoehn and Yahr scale; HO = Healthy Older adults; LOS = Limits of stability; M/L medial-lateral; MMSE = Mini-Mental State Examination; MS = Multiple sclerosis; Mth = Months; ORF = Older Adults At High Fall Risk; PD = Parkinson’s disease; PE = position error; PTA = percent of tactor activation; RMS = Symmetry Ratio; SBDT = Standard Balance Deficit Test; SBS = Smarter Balance System; SRT = Stepping reaction time; SOT = Sensory organization test; TUG = Time Up and GO test; VSS = vestibular symptom score; WSBE = Weight-shifting balance exercises; XCOR = cross-correlation; Yr = years.

**Table 2:** Modified NOS scale scores of the included studies.

First Author, Year	Study Type	Selection	Treatment Protocol	Outocome(s)	Total
Rossi, 2020 [32]	Proof-of-concept	*	**	***	6/7
Kodama, 2020 [34]	Clinical Trial	*	**	***	6/7
Afzal, 2018 [50]	Clinical Trial	*	*	***	5/7
Fung, 2018 [45]	Longitudinal Study	*	*	***	5/7
High, 2018 [44]	Clinical Trial	*	*	***	5/7
Lee, 2018 [46]	Clinical Trial	*	*	***	5/7
Yasuda, 2018 [54]	Clinical Trial	*	**	***	6/7
Van Wegen, 2018 [43]	Multiple case control pre-post design	*	*	**	4/7
Afzal, 2017 [51]	Clinical trial	*	**	***	6/7
Otis, 2016 [53]	Clinical trial	*	**	***	6/7
Afzal, 2015 [52]	Clinical trial	N.A.	*	***	4/7
Lee, 2015 [47]	Clinical Trial	*	**	***	6/7
Lee, 2013 [42]	Clinical Trial	*	**	**	5/7
Rossi-Izquierdo, 2013 [48]	Clinical Trial	*	**	***	6/7

Star (*) = item present; maximum 2 stars (**) for the Selection criteria, maximum 2 stars (**) for Treatment Protocol and maximum 3 stars (***) for Outcome criteria. N.A. = not applicable.

**Table 3:** PEDro scores of the included studies.

First Author, Year	Study Type	Random Allocation	Concealed Allocation	Baseline Comparability	Participant Blinding	Therapist Blinding	Assessor Blinding	Adequate Follow-up	Intention-to-Treat Analysis	Between-Group Comparison	Point Estimates and Variability	Total (0 to 10)
Yasuda, 2017 [55]	RCT	N	N	Y	N	N	N	Y	Y	Y	Y	5/10
van der logt 2016 [41]	RCT (crossover study)	N	Y	N	N	N	N	N	N	Y	Y	4/10
Nanhoe-Mahabier, 2012 [35]	RCT	Y	N	Y	Y	N	N	Y	Y	Y	Y	7/10
Basta, 2011 [49]	RCT	Y	N	Y	Y	Y	N	N	N	Y	Y	6/10

RCT = Randomized Controlled Trial; Y = Yes; N = No.

## 4. Discussion

A systematic review and meta-analysis were performed to investigate the effectiveness of the VF on balance and gait rehabilitation in patients with neurological diseases. Results of the present systematic review suggest that VF programs are safe and could represent a short-term beneficial intervention for neurological patients. However, it is difficult to generalize the results founded for the relatively few studies enrolled and for the heterogeneity of the interventions.

Protocols differ with respect to duration, required tasks, feedback localization, and type of vibrotactile devices.

In many studies [34,35,41,42,43,44,45,46,47,48,49,54,55], vibrotactile stimuli were used as negative feedback to facilitate a postural reaction of the patient during the motor task. This cognitive-motor task could favor the active involvement of the patients. Several studies [32,35,41,42,44,45,46,47,50,51,52,53,55] performed the balance assessment immediately pre–post VF stimulation, evaluating only the short-term effects. Five studies [34,43,48,49,54] evaluated the long-term effects of VF combined with a rehabilitation program, and two studies [48,54] included a three-month follow-up.

In most studies, the vibrotactile vibrating motors were applied at the lower back level, probably because of the proximity with the center of mass (COM) position during quiet standing [56]. Moreover, this area provides a large, readily accessible surface with a relatively uniform shape that can be conveniently used to accommodate the vibrotactile device [57]. Lower limb application was used in five studies in order to directly influence the activity of the spinal locomotion centers during the gait [58,59]. Concerning the head-mounted devices, as the authors themselves declared [35], they may be preferred over the other applications because the proximity of the cranial nerves to the cortical centers could eliminate potential errors and delays in sensory transmission and integration [60]. Despite this, the patients reported that they were more prompted to adjust the position of the head rather than the position of the whole body, as a result of this type of vibration.

VF was shown to be easily transmitted under clothing instead; no differences were found between the direct application of vibrating motors on the skin [46] or through a belt.

Due to the heterogeneity, it was not possible to detect if the application of the vibrotactile information in a determinate body area could be better than another one. 

Additionally, with respect to the type of device used, it is not possible to define a preferability, as eight studies used vibrotactile effectors of different shapes and sizes, self-produced or unspecified devices [34,42,50,51,52,54,55,56]. The commercial vibrotactile devices used were: WEARHAP-Pd device [32]; SBS (Smarter Balance System) [45]; Sensory Kinetic System [44]; UpRight [43]; Balance Freedom [41]; Vibrotactile circuit C2 tractors (Engineering Acoustic Inc.) [46,47]; Vibrotactile NFT and Vertiguard-RT [48] Vertiguard training device [49]. 

Changes in the level of displacements of the center of mass in static and dynamic position and changes during the gait were the most investigated outcomes. The instrumental assessment was carried out before the administration of the vibrotactile stimulus, during the execution of the motor tasks with VF application, and after. Few studies used clinical assessment of gait and balance. 

Four RCTs [35,41,49,55] showed that the use of VF during a rehabilitation program could be an efficient method to reduce the body sway in PD e SM patients. Moreover, its effectiveness seems possible for several types of gait and balance disorders [49]. Actually, the results obtained from meta-analysis largely support the effectiveness of VF training if compared to no feedback programs. However, some issues such as the small number of included studies, several differences in study protocols (mostly assessment procedures) and considerable levels of heterogeneity invite to caution in interpreting these data. Unfortunately, there are not enough clinical evaluations to enable the assessment of VF training on the patient’s quality of life and on daily living activity. However, it could be possible to speculate that the positive influence on the parameters of gait and balance shown by different studies, may also have a positive effect on these two not fully investigated aspects. In support of this, Rossi-Izquierdo et al. [48] showed that the group of patients with PD who performed the rehabilitation training supported by the VF had a significant reduction pre- and post-training in the standard balance deficit test (SBDT) composite score, associated with a significant improvement in Dizziness Handicap Inventory (DHI) and Activities-specific balance confidence scale (ABC) scores and associated with a significant reduction in the risk of falling. Moreover, Yasuda et al. [54] reported statistical significance in pre–post training analysis in Berg Balance Scale (BBS), Functional Reach Test (FRT), and in Timed-Up and Go test (TUG) in chronic hemiparetic stroke patients. Otis et al. [53] showed the use of VF as an enactive sole that uses a rhythmic vibrotactile cueing, while patients walked over different types of soil, could be useful in reducing the risk of falls in patients with PD. Another point in support of a possible positive implication on the quality of life of patients with PD of VF as supportive feedback in rehabilitation is given by Rossi et al. [32], who showed a reduction of the freezing of gate (FOG) duration. 

The results showed that the VF can influence walking and gait parameters of patients with neurological diseases immediately and after a rehabilitation program lasting a few days. The lack of significant results at follow up [48,49] could lead to hypothesize that the VF has a greater efficacy by wearing it and in the short time after treatment than in the long term. 

The positive effects of the VF in gait and balance parameters are reported to be greater if the vibratory stimulus is combined with other feedback as the haptic one [50,51,52] and the visual one [47]. 

The heterogeneity of the rehabilitation protocols and of the population does not make it possible to reach a conclusion on its effectiveness in rehabilitation but demonstrates the versatility of VF in different situations and conditions [49]. Furthermore, the heterogeneity of settings highlights the possibility to use VF by the patients independently, even in a domestic situation [45]. These characteristics make it a candidate to be considered as a supportive sensory stimulus in the context of the rehabilitation intervention focused on sensory-motor integration [55]. 

### 4.1. Strengths of the Systematic Review

To the best of the authors’ knowledge, this review is the first aimed at investigating the effectiveness of the VF on balance and gait rehabilitation in patients with neurological disease. The strengths of this systematic review are: (i) to have highlighted that the VF is safe and could easily implement a standard rehabilitation program in patients affected by neurological disease; (ii) our methods were based on Preferred Reporting Items for Systematic reviews to minimize potential sources of bias; and (iii) inclusion and exclusion criteria were defined to minimize selection bias. 

### 4.2. Study Limitations

Several limitations in the present review and meta-analysis are acknowledged. Firstly, the small number of available RCTs precludes the possibility of comparing the rehabilitative approach supported by the VF with other types of treatments. Secondly, methodological heterogeneity (e.g., study designs, outcome measures) restricted the number of studies eligible for the quantitative analysis. In addition to this, data reporting was frequently incomplete or not always provided in a useful way to perform meta-analysis; when possible, information was obtained by contacting authors via e-mail, or conversely, we properly managed them on the basis of available data.

Thirdly, the variability of the interventions does not allow to identify a single rehabilitative protocol that verifies the effectiveness. Moreover, the number of patients for each included pathology is small. The internal validity of studies is also limited, and the methodological quality is low to medium as a consequence of the study designs: lack of randomization and blinding, small or uncontrolled groups. Furthermore, a clinical and instrumental assessment of motor abilities should have been carried out to better clarify the effects on dynamic postural stability and gait parameters [61,62,63].

## 5. Conclusions

This review and meta-analysis highlights the effects of the VF provided during motor tasks, on gait and balance in patients with neurological diseases. Although the VF could be considered in the context of neurorehabilitation as a supportive sensory stimulus, not enough trials were found to establish the effectiveness of a rehabilitation program that includes the VF. Clinical practices recommendation on its efficacy cannot be made due to the lack of high-quality studies and heterogeneity of treatment protocols. Further studies focused on the evaluation of the VF effects on the quality of life and daily living changes are recommended.

Neurophysiological mechanisms linking VF intervention to enhanced balance functions should be explored after interventions to investigate possible neural mechanisms underlying the vibrotactile-induced improvements.

High-quality RCTs with cost-effective and long-term evaluations are necessary to influence clinical practice and the decision making process in neurorehabilitation.

This review may be considered as a starting point for future RCTs that could investigate the effectiveness of a vibrotactile training on balance, gait, daily life activities, and on the quality of life of patients with neurological and cerebrovascular diseases. 

## Data Availability

Not applicable.

## Appendix A

### Appendix A.1. Search Strategy in MEDLINE (PubMed)

(“balance” AND “vibrotactile” OR “haptic” AND “neurological disorder*” OR “stroke” OR “parkinson” OR “multiple sclerosis” OR “traumatic brain injury”).

### Appendix A.2. Search Strategy in PEDro

- balance AND vibrotactile
- balance AND haptic
